# Cryopreservation of vegetative cells and zygotes of the multicellular volvocine green alga *Gonium pectorale*

**DOI:** 10.1186/s12866-022-02519-9

**Published:** 2022-04-14

**Authors:** Hisayoshi Nozaki, Fumi Mori, Yoko Tanaka, Ryo Matsuzaki, Haruyo Yamaguchi, Masanobu Kawachi

**Affiliations:** 1 Biodiversity Division, National Institute for Environmental Studies, Tsukuba, Ibaraki 305-8506 Japan; 2 Department of Biological Sciences, Graduate School of Science, The University of Tokyo, Bunkyo-ku, Tokyo, 113-0033 Japan; 3 Global Environmental Forum, Inarimae, Ibaraki 305-0061 Tsukuba-shi, Japan; 4 Faculty of Life and Environmental Sciences, University of Tsukuba, Tsukuba, Ibaraki 305-8572 Japan

## Abstract

**Background:**

Colonial and multicellular volvocine green algae have been extensively studied recently in various fields of the biological sciences. However, only one species (*Pandorina morum*) has been cryopreserved in public culture collections.

**Results:**

Here, we investigated conditions for cryopreservation of the multicellular volvocine alga *Gonium pectorale* using vegetative colonies or cells and zygotes. Rates of vegetative cell survival in a *G. pectorale* strain after two-step cooling and freezing in liquid nitrogen were compared between different concentrations (3% and 6%) of the cryoprotectant N,N-dimethylformamide (DMF) and two types of tubes (0.2-mL polymerase chain reaction tubes and 2-mL cryotubes) used for cryopreservation. Among the four conditions investigated, the highest rate of survival [2.7 ± 3.6% (0.54–10%) by the most probable number (MPN) method] was obtained when 2.0-mL cryotubes containing 1.0 mL of culture samples with 6% DMF were subjected to cryogenic treatment. Using these optimized cryopreservation conditions, survival rates after freezing in liquid nitrogen were examined for twelve other strains of *G. pectorale* and twelve strains of five other *Gonium* species. We obtained ≥ 0.1% MPN survival in nine of the twelve *G. pectorale* strains tested. However, < 0.1% MPN survival was detected in eleven of twelve strains of five other *Gonium* species. In total, ten cryopreserved strains of *G. pectorale* were newly established in the Microbial Culture Collection at the National Institute for Environmental Studies. Although the cryopreservation of zygotes of volvocine algae has not been previously reported, high rates (approximately 60%) of *G. pectorale* zygote germination were observed after thawing zygotes that had been cryopreserved with 5% or 10% methanol as the cryoprotectant during two-step cooling and freezing in liquid nitrogen.

**Conclusions:**

The present study demonstrated that cryopreservation of *G. pectorale* is possible with 6% DMF as a cryoprotectant and 1.0-mL culture samples in 2.0-mL cryotubes subjected to two-step cooling in a programmable freezer.

**Supplementary Information:**

The online version contains supplementary material available at 10.1186/s12866-022-02519-9.

## Background

The volvocine green algae are a model lineage for studying multicellularity and the evolution of sexes; extensive research in various areas of biology has been conducted using colonial or multicellular members of this lineage [1–4]. However, serious problems arise from culture strain maintenance using growing cultures; for example, during the long-term maintenance of cultures through subculturing, the inducibility of sexual reproduction and the ability to perform normal morphogenesis gradually decrease in multicellular volvocine species [5, 6]. Thus, long-term culture-maintained strains of these algae may not be suitable for studies of morphology and sexual reproduction. In addition, the maintenance of algal strains through subculturing under optimal or suboptimal conditions carries a high cost in both public culture collections and private laboratories. Therefore, cryopreservation protocols are greatly needed for culture strains of colonial or multicellular volvocine species. Among public culture collections of algae worldwide, however, only four cryopreserved strains of a single multicellular volvocine species (*Pandorina morum*) are present in the Culture Collection of Algae at the University of Texas at Austin (https://utex.org/). This situation may be influenced by low recovery rates or difficulty of cryopreservation of the multicellular volvocine algae due to their large reproductive cells and expanded extracellular matrix surrounding the cells [2].

Mori et al. [7] examined survival after exposure to liquid nitrogen freezing conditions in seventy-six strains of ten multicellular volvocine genera maintained in the Microbial Culture Collection at the National Institute for Environmental Studies (MCC-NIES) (https://mcc.nies.go.jp/index_en.html [8]) using dimethyl sulfoxide (DMSO) as a cryoprotectant, but only one of eighteen strains of *Gonium* was able to survive freezing.

Although Nakazawa and Nishii [9] reported the recovery of growth after cryopreservation in various multicellular volvocine species when amidic and acetonic cryoprotectants were used, no details of survival after cryopreservation were reported. Multicellular volvocine species generally produce thick-walled zygotes that are resistant to dry and cool conditions [2]. However, the cryopreservation of zygotes of colonial or multicellular volvocine species has not been studied previously.

**Fig. 1:**
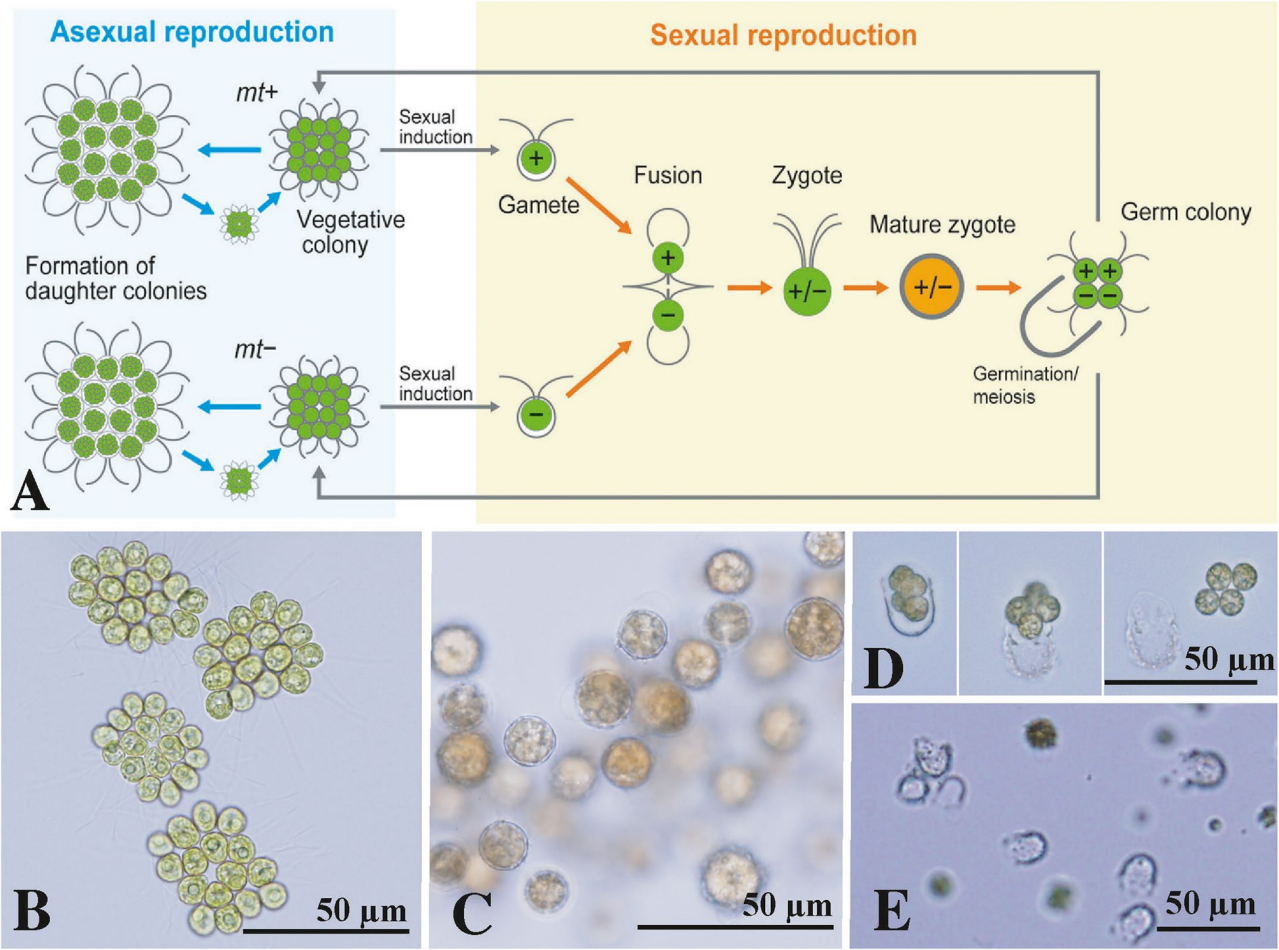
Life cycle of *Gonium pectorale*. **A** Diagram of life cycle showing facultative sexual reproduction and heterothallic mating system. Based on Nozaki & Ito [21] and the present study. **B** Vegetative colonies of NIES-4502. **C** Mature zygotes ready for germination (NIES-4501 × NIES-4502). **D** Successive stages of zygote germination showing release of a four-celled germ colony. All at same magnification. **E** Empty zygote walls after germination (NIES-4501 × NIES-4502)

The present study was undertaken to establish methods for the cryopreservation of culture strains of multicellular volvocine algae, focusing on the simple multicellular volvocine species *Gonium pectorale* (Fig. 1). *G. pectorale* is a heterothallic species that was recently studied using whole-genome sequencing to resolve the genetic basis of the transition to multicellularity and the evolution of sex [10, 11]. Methods for the cryopreservation of *G. pectorale* were explored using vegetative cells and zygotes in this study.

## Materials and methods

### Culture strains used

New strains established from germinating zygotes of volvocine algae generally exhibit active sexual reproduction [5]. Therefore, we used four new F1 strains of *G. pectorale* obtained from the germination of dried zygotes of the original or parental strains (Kaneko3 [NIES-1710] and Kaneko4 [NIES-1711]) of *G. pectorale* strains (K3-F3-4 [NIES-2863] and K4-F3-4) that have been used in previous genomic studies [10, 11]. The cultures were maintained in screw-cap tubes (18 × 150 mm) containing 10 mL artificial freshwater-6 (AF-6) or *Volvox* thiamin acetate (VTAC) medium [8] at 25 °C with a 12-h:12-h light:dark schedule under cool-white fluorescent lamps at an intensity of 100–130 μmol m^−2^ s^−1^. These new F1 strains (2021–0414-F1GP-2, 3, 7 and 8) will be available from MCC-NIES as NIES-4499–NIES-4502 (Additional file 1: Table S1). To determine the mating types of the new *G. pectorale* strains, the presence or absence of the mating type minus-specific minus dominance (*MID*) and mating type plus-specific gamete adhesion (*FUS1*) genes [11] was examined through genomic polymerase chain reaction (PCR) analyses using *MID*- and *FUS1*-specific primers (Additional file 1: Table S2, Figure S1). PCR was conducted as described previously [12] using disrupted cell solution as template DNA, along with KOD One PCR Master Mix (Toyobo, Osaka, Japan).

In addition to these four newly established strains of *G. pectorale*, nine axenic strains of *G. pectorale* [8] and twelve strains of five other *Gonium* species (Additional file 1: Table S1) were obtained from MCC-NIES. They were cultured in VTAC medium or USVT medium (VTAC medium supplemented with 40 mg/L urea and 40 mL/L soil extract [13] (Additional file 1: Table S1) at 25 °C with a 12-h:12-h light:dark schedule, then subjected to cryopreservation.

### Cryopreservation

To assess the optimal cryopreservation conditions for *G. pectorale*, we used N,N-dimethylformamide (DMF) as a cryoprotectant; Nakazawa and Nishii [9] previously demonstrated possible survival of *G. pectorale* vegetative cells after freezing in liquid nitrogen with 3% DMF. Because Nakazawa and Nishii [9] studied the cryopreservation of multicellular volvocine algae using 0.25-mL PCR tubes, we used similar PCR tubes that were pre-sterilized (0.2-mL 8-Strip PCR Tube with Attached Dome Cap, Sterilized, Clear, Neptune Scientific, San Diego, CA, USA). We also examined cryopreservation results when using 2-mL cryotubes (Cryo.s, 2-mL, Round Bottom, Starfoot Base, Greiner Bio-One, Kremsmünster, Austria), which are generally used for the cryopreservation of microalgae in MCC-NIES [7, 8, 14]. Thus, four types of cryopreservation conditions were examined using vegetative cells of *G. pectorale* strain 2021–0414-F1GP-8 (NIES-4502): 0.2-mL sample with 3% DMF in a 0.2-mL PCR tube, 0.2-mL sample with 6% DMF in a 0.2-mL PCR tube, 1.0-mL sample with 3% DMF in a 2.0-mL cryotube, and 1.0-mL sample with 6% DMF in a 2.0-mL cryotube. For cryopreservation, an actively growing, 2-day-old culture (approximately 10^6^ cells/mL) in VTAC medium (2–4 mL) was mixed with an equal volume of VTAC medium containing 6% or 12% DMF to prepare a sample with 3% or 6% DMF, respectively. The cells were exposed to the cryoprotectant at room temperature (20–25 °C) for 15 min. Then, 0.2 mL or 1.0 mL of the culture sample with DMF was transferred to a 0.2-mL PCR tube or 2-mL cryotube, respectively; the sample was subjected to two-step cooling using a programmable freezer (Controlled Rate Freezer, KRYO 560–16, Planer, Middlesex, UK) and liquid nitrogen [7, 14]. The cell suspensions in tubes were frozen in vapor phase of liquid nitrogen at cooling rate of –1 °C/minute to –40 °C. After 15 min of maintenance at –40 °C, the cell suspensions were cooled rapidly to –196 °C by immersion in liquid nitrogen, and finally stored at –190 °C in vapor phase of liquid nitrogen. To assess the viability of cells frozen in liquid nitrogen, the frozen samples in tubes were thawed in a 40 °C water bath while the tube was shaken by hand until the ice crystals disappeared (approximately 30 or 120 s in 0.2-mL PCR tube or 2-mL cryotube, respectively); then, 0.1 mL of the diluted sample (0.05 mL melted sample plus 0.05 mL fresh medium) was immediately subjected to analysis using the most probable number (MPN) method [14–16]. For the MPN method, eight wells in each dilution series of a 48-well microplate (Cellstar Cell Culture Multiwell Plate with Lid, Greiner Bio-One) were filled with 0.9 mL of growth medium. Three replicates of eight 1/10th dilutions were performed for each cryotube or PCR tube of sample using a 6-channel pipette (Pipet-Lite Adjustable Spacer LA6-1200XLS, Mettler-Toledo, Greifensee, Switzerland). As a control, three replicates of eight 1/10th dilutions of cultures without cryogenic treatment and cryoprotectant were treated in the same manner. The plates were initially incubated in darkness at 25 °C for 2 days, then transferred to a 12-h:12-h light:dark schedule at 25 °C for 2 weeks. This initial dark incubation has been shown to effectively improve the survival rates of many microalgal strains; it has become routine procedure for cryopreservation at MCC-NIES. Each well was scored for growth and MPN values (cell numbers) were estimated based on those scores using MPN Calculator 3.1 < https://softdeluxe.com/MPN-Calculator-444229/ > [17, 18]. The recovery rate of viable cells (%) was calculated relative to the viable cell count in the unfrozen control using the MPN method. For each of the four types of cryopreservation conditions, recovery rates were measured based on six tubes from two independent experiments (Table 1).

For the cryopreservation of zygotes of *G. pectorale*, “zygotes ready for germination” were prepared. Approximately 1-week-old cultures in VTAC medium (5 mL each) of two complementary mating types (NIES-4501 and NIES-4502, Additional file 1: Table S1) were mixed in Petri dishes (57 × 16 mm) (60 mm/non-treated Dish, IWAKI AGC Techno Glass, Shizuoka, Japan) to produce mature zygotes. After approximately 1 week, mature zygotes had formed; these were subjected to dark treatment on 1% agar plates (AF-6 medium) for at least 30 days at 20 °C. Such zygotes (ready for germination after dark treatment) began germination within 48 h after transfer to liquid VTAC medium under a 12-h:12-h light:dark cycle at 25 °C. The zygotes ready for germination were suspended in liquid VTAC medium and mixed with cryoprotectant in VTAC medium to prepare a 0.1-mL sample (approximately 10^5^ zygotes/mL) in a 0.2-mL PCR 8-strip tube; the sample was subjected to two-step cooling and freezing in liquid nitrogen, as described above for the cryopreservation of vegetative cells. For viability measurement, the frozen tube was thawed at 40 °C in a water bath and the melted mixture (0.1 mL) was inoculated into 10 mL VTAC medium in a 6-well plate (PS, with Lid, Greiner Bio-One); it was initially grown in darkness for 2 days and then under a 12-h:12-h light:dark cycle at 25 °C for 2 days. Subsequently, the survival of zygotes was assessed based on germination rates via light microscope examination through the direct counting of empty zygote walls after germination and intact zygotes (at least 46 zygotes or zygote walls were counted for each PCR tube). Three types of cryoprotectants (DMSO, methanol, and DMF) were examined with respect to zygote survival after cryopreservation. We compared results among six cryopreservation conditions: 0.1-mL sample with 5% DMSO in a 0.2-mL PCR tube, 0.1-mL sample with 10% DMSO in a 0.2-mL PCR tube, 0.1-mL sample with 5% methanol in a 0.2-mL PCR tube, 0.1-mL sample with 10% methanol in a 0.2-mL PCR tube, 0.1-mL sample with 5% DMF in a 0.2-mL PCR tube, and 0.1-mL sample with 10% DMF in a 0.2-mL PCR tube. For each of these conditions, the germination rates were measured based on three different tubes.

## Results

### Cryopreservation of vegetative cells

Table 1 shows MPN results for the survival rates of cryopreserved vegetative cells of *G. pectorale* strain NIES-4502 after four types of cryogenic treatment. A significant difference (*p* < 0.05) was detected in the recovery rate between the two types of tubes, but not between 3 and 6% DMF. Among the four conditions investigated, the MPN survival rate was highest [2.7 ± 3.3% (0.65–10%)] with 6% DMF in 2.0-mL cryotubes (Table 1).

**Table 1:** Comparison of results of four types of cryopreservation conditions for vegetative cells of *Gonium pectorale* strain NIES-4502 based on most probable number (MPN) methods

		Experiment I	Experiment II
Conditions for cryopreservation	Total viability^**a**^ (range) [%]	MPN cell numbers in three tubes (/mL) (control)^b^	MPN cell numbers in three tubes (/mL) (control)^b^
0.2 mL 3% DMF in 0.2 mL PCR tube	0.31 ± 0.67 (0.0016–1.8)	7,2, 7.2, 7.2 (46,000)	7.2, 18, 8400 (460,000)
0.2 mL 6% DMF in 0.2 mL PCR tube	0.38 ± 0.36 (0.031–1.0)	460, 460, 840 (84,000)	150, 220, 300 (460,000)
1.0 mL 3% DMF in 2 mL cryotube	2.1 ± 2.2 (0.04–10)	300, 840, 3000 (46,000)	180, 1800, 15,000 (460,000)
1.0 mL 6% DMF in 2 mL cryotube	2.7 ± 3.3 (0.65–10)	460, 1500, 8400 (84,000)	3000, 7600, 8400 (460,000)

^a^ Significant difference (*p* < 0.05) was detected between two types of tubes based on unweighted-mean ANOVA analyzed by js-STAR XR release 1.6.6j < http://www.kisnet.or.jp/nappa/software/star/index.htm >

^b^ Numbers corrected based on “0.05 mL of the sample plus 0.05 mL of VTAC medium” used for initial inoculation (see Materials and methods)

Thus, recovery rates based on the MPN method after the cryopreservation of twelve other strains of *G. pectorale* were examined using 6% DMF and 2-mL cryotubes, as described for *G. pectorale* strain NIES-4502, with modification of the volume (0.1 mL) of the first inoculum from the cryotubes and use of the USVT medium for two strains (Additional file 1: Table S1). In addition, immediately after thawing of the three frozen cryotubes of each strain, 0.5 mL of the melted sample in each cryotube was inoculated into fresh growth medium (10 mL) in a screw-cap culture tube (first inoculation); subsequently, 0.5 mL of the first inoculation were transferred to another 10 mL of fresh growth medium (second inoculation) to confirm the recovery of frozen and thawed cells in the culture tubes used in MCC-NIES. Based on the optimized cryopreserved conditions (1.0 mL with 6% DMF in 2-mL cryotubes, Table 2), ≥ 0.1% MPN viability rates after freezing in liquid nitrogen and thawing were calculated for nine of the twelve *G. pectorale* strains (Table 2). Each of the nine *G. pectorale* strains plus strain NIES-4502 (Table 1) exhibited active growth in 10-mL cultures from three cryotubes after the first and second inoculations (Table 1). Thus, ten cryopreserved strains of *G. pectorale* were newly established in MCC-NIES.

**Table 2:** Comparison of recovery results of twelve strains of *G. pectorale* (Additional file 1: Table S1) after possible optimal cryogenic treatment (1.0 mL 6% DMF in 2 mL cryotube; Table 2) in liquid nitrogen

Strain designation	Survivability based on MPN method [%]	Number of viable cultures by 1st [2nd] inoculation with 3 [3] 10 mL cultures
NIES-4499	1.7 ± 1.7	3 [3]
NIES-4500	0.19 ± 0.05	3 [3]
NIES-4501	0.1 ± 0.0	3 [3]
NIES-4502	2.7 ± 3.3 (Table 1)	3 [3]
NIES-2261 ^a^	0.0018 ± 0.0008	3 [3]
NIES-2262	0.10 ± 0.046	3 [3]
NIES-4121	3.3 ± 1.3	3 [3]
NIES-468	0.28 ± 0.19	3 [3]
NIES-469 ^a^	0.0013 ± 0.0023	2 [0]
NIES-569	0.28 ± 0.19	3 [3]
NIES-570 ^a^	0.012 ± 0.008	3 [3]
NIES-645	4.2 ± 8.5	3 [3]
NIES-646	38 ± 28	3 [3]

^a^ Not used for cryopreserved strain in MCC-NIES because of < 0.1% MPN survivability

For twelve strains of five other *Gonium* species (Additional file 1: Table S1), we used the same cryogenic treatment conditions (6% DMF and 2.0-mL cryotubes) that were effective for the cryopreservation of *G. pectorale* (Table 2). However, ≥ 0.1% MPN survival was detected in only a single strain (*Gonium viridistellatum* strain NIES-288) and active growth in all six 10-mL cultures was not observed for each strain with two successive inoculations after freezing in liquid nitrogen and thawing (Table 3).

**Table 3:** Comparison of results of strains of five other species of *Gonium* (Additional file 1: Table S1) after possible optimal cryogenic treatment (1.0 mL 6% DMF in 2 mL cryotube; Table 1) in liquid nitrogen

Species	Strain designation	Survivability based on MPN method [%]	Number of viable cultures by 1st [2nd] inoculation with 3 [3] 10 mL cultures
*G. maiaprilis*	NIES-2455	0.00043 ± 0.00075	1[0]
	NIES-2456	0	0[0]
*G. multicoccum*	NIES-737	0	0[0]
	NIES-885	0	1[0]
*G. octonarium*	NIES-851	0.054 ± 0.042	3[1]
	NIES-852	0.0035 ± 0.049	3[0]
*G. quadratum*	NIES-652	0	0[0]
	NIES-653	0	1[0]
*G. viridistellatum*	NIES-288	0.13 ± 0.23	3[1]
	NIES-290	0.0022 ± 0.0019	3[1]
	NIES-654	0	0[0]
	NIES-655	0	0[0]

### Cryopreservation of zygotes

**Fig. 2:**
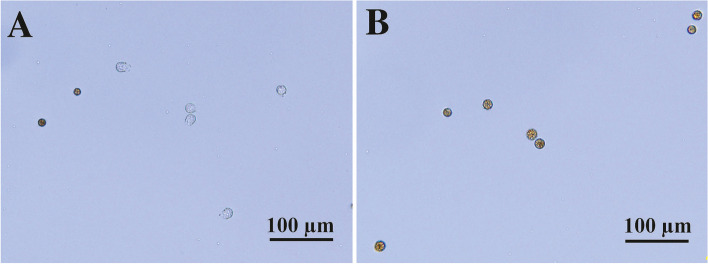
Effects of cryoprotectants on germination of *Gonium pectorale* zygotes (NIES-4501 × NIES-4502) that were subjected to liquid nitrogen freezing (Table 4). **A** 5% methanol cryogenic treatment. Note empty zygote walls after germination. **B** 5% DMF cryogenic treatment. Note intact, walled zygotes

Among the three cryoprotectants used for zygotes, methanol was most effective for supporting high germination rates among *G. pectorale* zygotes after freezing in liquid nitrogen and thawing (Table 4). Approximately 60% of cryo-treated zygotes germinated when methanol was used as the cryoprotectant (Fig. 2A), compared with approximately 60–80% of unfrozen zygotes (controls) (Table 4). In contrast, only 0–20% of zygotes germinated when other cryoprotectants (DMSO and DMF) were used (Fig. 2B).

**Table 4:** Comparison of zygote germination rates of *Gonium pectorale* strains NIES-4501 × NIES-4502 after cryopreservation among six types of cryoprotectant conditions

Cryoprotectant	Zygote germination rate (rates in three PCR tubes) [%]	Control zygote germination rate^a^ [%]
5% DMSO	2.9 ± 2.9 (2.8, 5.8, 0)^b^	77
10% DMSO	26 ± 7.9 (26, 19, 34)^b^	72
5% Methanol	61 ± 8.7 (70, 52, 60)^b^	67
10% Methanol	59 ± 2.0 (61, 57, 60)^b^	61
5% DMF	0.42 ± 0.73 (1.3, 0, 0)^b^	76
10% DMF	5.9 ± 5.2 (12, 3.1, 2.7)^b^	69
No cryoprotectant	0.0 ± 0.0 (0, 0, 0)	78

^a^ Without cryopreservation

^b^ Significant difference (*p* < 0.01) was detected between three types of cryoprotectants based on unweighted-mean ANOVA analyzed by js-STAR XR release 1.6.6j < http://www.kisnet.or.jp/nappa/software/star/index.htm >

**Fig. 3:**
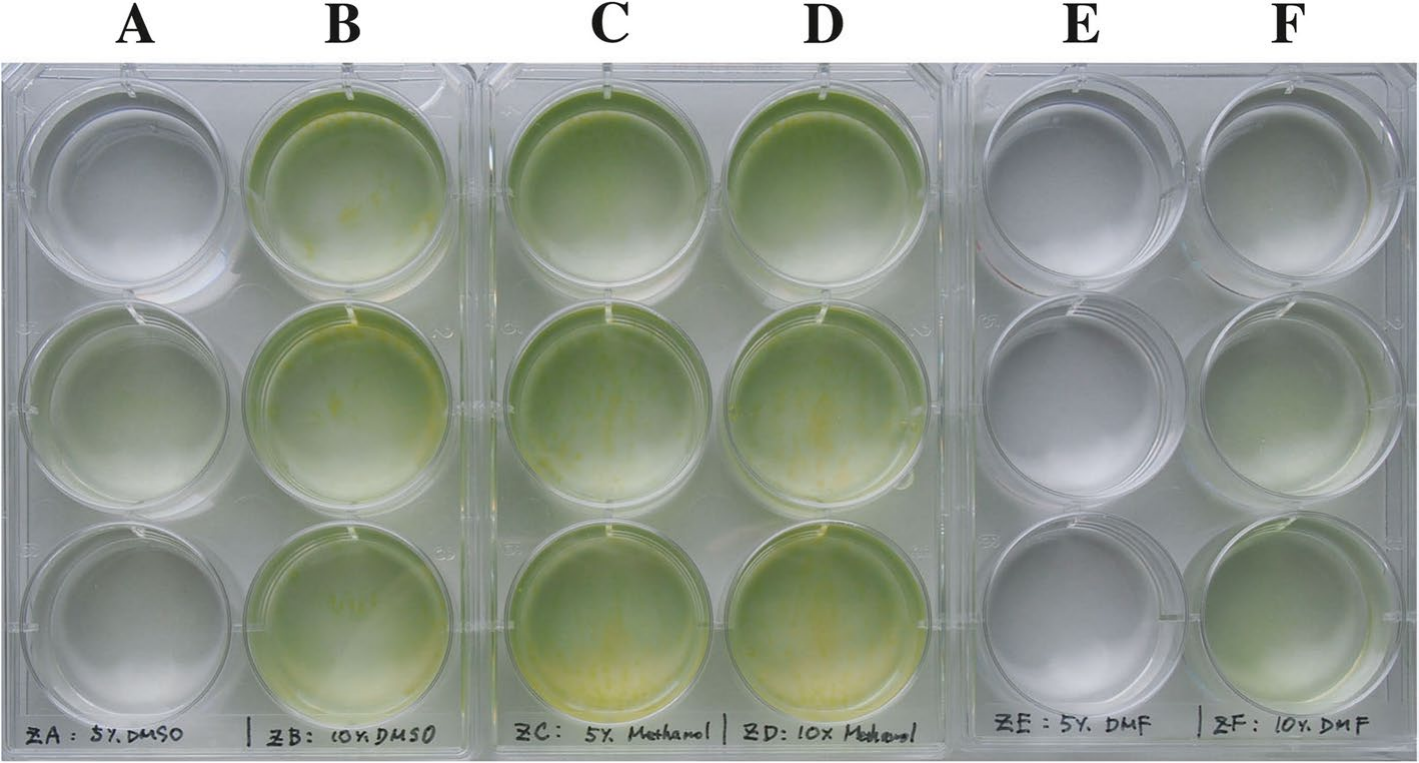
Five-day-old growth of *Gonium pectorale* progenies given rise from zygotes (NIES-4501 × NIES-4502) that were subjected to liquid nitrogen freezing with six types of cryoprotectants. Compare these results with zygote germination rates (Table 4). **A** 5% DMSO. **B** 10% DMSO. **C** 5% methanol. **D** 10% methanol. **E** 5% DMF. **F** 10% DMF

Irrespective of cryogenic treatment, new cells originating from the zygotes appeared to grow normally, because the degree of greenish color in the cultures after 5 days (Fig. 3) was consistent with the calculated zygote germination rates (Table 4).

## Discussion

Mori et al. [7] showed that only one of the eighteen strains of *Gonium* deposited in NIES-MCC survived after two-step cooling and freezing in liquid nitrogen using DMSO as a cryoprotectant. However, we found that 77% (10/13) of axenic strains of *G. pectorale* in MCC-NIES could be cryopreserved based on a threshold of ≥ 0.1% MPN survival and recovery of active growth in all six 10-mL cultures after two successive inoculations of liquid nitrogen-frozen cultures using 6% DMF and 2.0-mL cryotubes (Table 2). Nakazawa and Nishii [9] demonstrated the survival of *G. pectoral*e strain NIES-1710 (Kaneko3) with 3% DMF as a cryoprotectant in a 0.25-mL PCR tube, which enabled easier rewarming and thawing. However, the present study demonstrated that the survival rates of frozen and thawed *G. pectorale* vegetative cells were lower in 0.2-mL PCR tubes than in 2.0-mL cryotubes (Table 1). These results conflict with the fact that rapid thawing of small-volume sample enhances recovery rates of cryopreserved cells [9, 19]. The higher recovery rates obtained using cryotubes (1.0 mL sample) than using PCR tubes (0.2 mL sample) in the present study (Table 1), however, may be explained by difference in material or synthetic resin (polypropylene) between the two types of tubes; PCR tubes are designed for effective PCR reaction in tubes, whereas cryotubes are manufactured for maintaining living cells with cryoprotectant in the tube. Some chemical compounds leached from the PCR tube may inhibit recovery or growth of living green algal cells. Even if the materials of both types of the tubes are the same, the effects of the inner surface of the tubes (chemical compounds leaching from the polypropylene tubes in the presence of cryoprotectant during freezing and thawing) on the samples are larger in a small PCR tube than in a large cryotube. In addition, our use of a programmable freezer for two-step freezing in liquid nitrogen may result in successful recovery of cryopreserved vegetative cells of *G. pectorale*. Nakazawa and Nishii [9] used a preservation module (StrataCooler® Cryo Lite, Stratagene, La Jolla, CA, USA) that is kept in a deep freezer (–80 °C) to cool cells prior to storage in liquid nitrogen. Thus, we also examined recovery rates of *G. pectorale* vegetative cells cryopreserved with 6% DMF by two-step freezing in liquid nitrogen using a similar preservation module (Thermo Scientific™ Mr. Frosty™ Freezing Container) that can provide freeze cells by achieving a rate of cooling very close to -1 °C/minute within a deep freezer. However, extremely low survivability (< 0.004%) was obtained in both types of tubes by using the preservation module for two-step cooling and freezing in liquid nitrogen (Additional file 1: Table S3).

Although DMF is an effective cryoprotectant that supports robust survival of cryopreserved vegetative cells of *G. pectorale* (Tables 1 and 2), it is inappropriate for zygotes of this species. Among the three cryoprotectants examined in the present study, ethanol provided high germination and viability rates (approximately 60%) of cryo-treated zygotes (Table 4). Thus, the most effective cryoprotectant may differ among cells at different life cycle stages or with different cell structures (i.e., biflagellate vegetive cells within a gelatinous matrix versus thick-walled immobile zygotes) (Fig. 1). The zygotes of volvocine green algae form for survival during cool and dry seasons in natural habitats [2]; drying may thus be an appropriate preservation method for volvocine zygotes. However, the viability of dried algal cells generally decreases over time [20] and the germination rates of long-term-maintained (21-year-old) dried zygotes of *G. pectorale* in our laboratory were low (less than 5%; see Materials and methods). Nevertheless, the cryopreserved zygotes of *G. pectorale* support almost permanent maintenance of zygotes with high germination rates (Table 4). When such cryopreserved zygotes are used for further experimental studies, the isolation of a single vegetive colony or cell after zygote germination is necessary to establish clonal cultures. Determination of mating type, plus or minus, of such newly established strains can be conducted using genomic PCR with primers for mating type-specific genes (Additional file 1: Table S2).

## Conclusion

The present study demonstrated that cryopreservation of vegetative cells of the multicellular volvocine green algal species *G. pectorale* was generally successful using 6% DMF and actively growing cultures in 2.0-mL cryotubes that were subjected to two-step cooling in a programmable freezer (Tables 1 and 2). However, this protocol was not appropriate for vegetative cells of other species in the genus *Gonium* (Table 3) or zygotes of *G. pectorale* (Table 4). Vegetative cells of other multicellular volvocine algal species (e.g., *Pleodorina starrii*, *Volvox barberi*, and *Volvox gigas*) do not survive two-step cooling and freezing in liquid nitrogen with 3% DMF [9]. However, ethanol is a good cryoprotectant for vegetative cells of *Pandorina* [9] (https://utex.org/) and zygotes of *G. pectorale* (Table 4), although the effects of DMF on the cryopreservation of *Pandorina* vegetative cells have not been reported. Thus, further studies regarding the cryopreservation of multicellular volvocine algae using various cryogenic treatments are needed to establish more cryopreserved strains of such algal genera in public culture collections.

## Supplementary Information

**Additional file 1: Table S1.** List of strains of *Gonium* used in this study. **Table S2.** Specific primers used for genomic PCR for strains of *Gonium pectorale*. **Table S3.** Recovery results of vegetative cells of *Gonium pectorale* strain NIES-4502 after possible optimal cryogenic treatments (6% DMF; Table 1) in liquid nitrogen by using a simple cryopreservation module (Thermo Scientific™ Mr. Frosty™ Freezing Container^a^, Thermo Fisher Scientific, Waltham, MA, USA) for two-step cooling in cryopreservation^b^. ** Figure S1.** Mating type determination of four newly established strains of *Gonium pectorale* (NIES-4499–4502, Table 1) by genomic PCR of mating type minus-specific minus dominance gene (*MID*) and mating type plus-specific gamete plasma membrane protein gene (*FUS1*). **Figure S2.** Full length, unprocessed gel images of the three genes shown in Figure S1.

## Data Availability

All data generated or analyzed during this study are included in this published article and its additional file.

## Abbreviations

AF-6: Artificial freshwater-6
CCA-UTEX: Culture Collection of Algae at the University of Texas at Austin
DMF: N,N-dimethylformamide
DMSO: Dimethyl sulfoxide
MPN: The most probable number
MCC-NIES: Microbial Culture Collection at the National Institute for Environmental Studies
VTAC: Volvox thiamin acetate
