# Correction: Cryopreservation of vegetative cells and zygotes of the multicellular volvocine green alga *Gonium pectorale*

**DOI:** 10.1186/s12866-022-02539-5

**Published:** 2022-05-18

**Authors:** Hisayoshi Nozaki, Fumi Mori, Yoko Tanaka, Ryo Matsuzaki, Haruyo Yamaguchi, Masanobu Kawachi

**Affiliations:** 1 Biodiversity Division, National Institute for Environmental Studies, Tsukuba, Ibaraki, 305-8506 Japan; 2 Department of Biological Sciences, Graduate School of Science, The University of Tokyo, Bunkyo-ku, Tokyo, 113-0033 Japan; 3 Global Environmental Forum, Inarimae, Ibaraki, Tsukuba-shi 305-0061 Japan; 4 Faculty of Life and Environmental Sciences, University of Tsukuba, Tsukuba, Ibaraki, 305-8572 Japan

**Correction: BMC Microbiol 22, 103 (2022)**

**https://doi.org/10.1186/s12866-022-02519-9**

Following the publication of the original paper [1], the authors spotted error in Additional file 1 (Table S3). Corrected file is captured as supplementary file of this article.

## Supplementary Information

**Additional file 1: Table S1**. List of strains of *Gonium* used in this study. **Table S2.** Specific primers used for genomic PCR for strains of *Gonium pectorale* (Additional file 1: Table S1). **Table S3.** Recovery results of vegetative cells of *Gonium pectorale* strain NIES-4502 after possible optimal cryogenic treatments (6% DMF; Table 1) in liquid nitrogen by using a simple cryopreservation module (Thermo Scientific™ Mr. Frosty™ Freezing Container, Thermo Fisher Scientific, Waltham, MA, USA) for two-step cooling in cryopreservation. **Figure S1**. Mating type determination of four newly established strains of *Gonium pectorale* (NIES-4499–4502, Additional file 1: Table S1) by genomic PCR of mating type minus-specific minus dominance gene (*MID*) and mating type plus-specific gamete plasma membrane protein gene (*FUS1*). *Actin* is an autosomal gene (control). For primers used, see Additional file 1: Table S2. A. NIES-4499. B. NIES-4500. C. NIES-4501. D. NIES-4502. For full-length original gel images, see Additional file 1: Figure S2. **Figure S2**. Full length, unprocessed gel images of the three genes shown in Additional file 1: Figure S1. Marker 6(λ/StyI digest)marker (NIPPON GENE, Tokyo, Japan) was used as a molecular size marker (1^st^, 6^th^, 11^th^ and 17^th^ lanes). 2^nd^, 3^rd^, 4^th^ and 5^th^ lanes: A, B, C and D, respectively, of *MID* (Additional file 1: Figure S1). 7^th^, 8^th^, 9^th^ and 10^th^ lanes: A, B, C and D, respectively, of *Actin* (Additional file 1: Figure S1). 12^th^, 13^th^, 14^th^ and 15^th^ lanes: A, B, C and D, respectively, of *FUS1* (Additional file 1: Figure S1).
