# Supplementary material for: Cryopreservation of vegetative cells and zygotes of the multicellular volvocine green alga Gonium pectorale
Source: BMC Microbiol. 2022 Apr 14;22:103. doi: 10.1186/s12866-022-02519-9 (PMC9008917; doi:10.1186/s12866-022-02519-9)
Supplement: Supplementary file 1 — Additional file 1: Table S1. List of strains of Gonium used in this study. Table S2. Specific primers used for genomic PCR for strains of Gonium pectorale. Table S3. Recovery results of vegetative cells of Gonium pectorale strain NIES-4502 after possible optimal cryogenic treatments (6% DMF; Table 1) in liquid nitrogen by using a simple cryopreservation module (Thermo Scientific™ Mr. Frosty™ Freezing Containera, Thermo Fisher Scientific, Waltham, MA, USA) for two-step cooling in cryopreservationb. Figure S1. Mating type determination of four newly established strains of Gonium pectorale (NIES-4499–4502, Table 1) by genomic PCR of mating type minus-specific minus dominance gene (MID) and mating type plus-specific gamete plasma membrane protein gene (FUS1). Figure S2. Full length, unprocessed gel images of the three genes shown in Figure S1. [file 12866_2022_2519_MOESM1_ESM.pdf]

Table S1. List of strains of *Gonium* used in this study

Species	Strain designation	Mating type or sexuality	Date of establishment of strain	Growth medium
G. pectorale	NIES-4499 (2021-0414-F1GP-2 [a])	Mating type plus (without *MID*) [b]	2021 April	VTAC
	NIES-4500 (2021-0414-F1GP-3 [a])	Mating type minus (with *MID*) [b]	2021 April	VTAC
	NIES-4501 (2021-0414-F1GP-7 [a])	Mating type minus (with *MID*) [b]	2021 April	VTAC
	NIES-4502 (2021-0414-F1GP-8 [a])	Mating type plus (without *MID*) [b]	2021 April	VTAC
	NIES-2261	Sexually compatible with NIES-2262	1994 April	USVT
	NIES-2262	Sexually compatible with NIES-2261	1994 April	VTAC
	NIES-4121		2014 December	VTAC
	NIES-468	Sexually compatible with NIES-469	1979 April	USVT
	NIES-469	Sexually compatible with NIES-468	1979 April	VTAC
	NIES-569	Sexually compatible with NIES-570	1988 November	VTAC
	NIES-570	Sexually compatible with NIES-569	1988 November	VTACUS VT

	NIES-645	Sexually compatible with NIES-646	1985 September	VTAC
	NIES-646	Sexually compatible with NIES-645	1985 September	VTAC
G. maiaprilis	NIES-2455	Mating type plus (without *MID*) [c]	2007 April	VTAC
	NIES-2456	Mating type minus (with *MID*) [c]	2007 April	VTAC
G. multicoccum	NIES-737	Homothallic	1990 May	USVT
	NIES-885		1956 March	VTAC
G. octonarium	NIES-851	Mating type plus (without *MID*) [c]	Before 1998 [d]	VTAC
	NIES-852	Mating type minus (with *MID*) [c]	Before 1998 [d]	VTAC
G. quadratum	NIES-652	Mating type minus (with *MID*) [c]	1990 April	VTAC
	NIES-653	Mating type plus (without *MID*) [c]	1990 April	VTAC
G. viridistellatum	NIES-288	Mating type minus (with *MID*) [e]	1973-1977 [f]	VTAC
	NIES-290	Mating type plus (without *MID*) [e]	1973-1977 [f]	VTAC
	NIES-654	Mating type plus (without *MID*) [c]	1985 November	VTAC

NIES-655	Mating type minus (with *MID*) [c]	1985 November	USVT

[a] Newly established from dried zygotes of NIES-1710 x NIES-1711 in the present study.

[b] See Fig. 2.

[c] Based on Hamaji et al. [1].

[d] Strains provided by Dr. Richard C. Starr (1924-1998).

[e] Based on Hamaji et al. [1] and Nozaki [2].

[f] Strains established between field collection (1973) and description of this species (1977) [3].

References

1. Hamaji T, Ferris PJ, Nishii I, Nishimura Y, Nozaki H. Distribution of the Sex-Determining Gene MID and Molecular Correspondence of mating types within the isogamous genus *Gonium* (Volvocales, Chlorophyta). PLoS One. 2013;8:e64385. doi: 10.1371/journal.pone.0064385.
2. Nozaki H. Morphological variation and reproduction in *Gonium viridistellatum* (Volvocales, Chlorophyta). Phycologia. 1989;28:77–88. doi: 10.2216/i0031-8884-28-1-77.1.
3. Watanabe M. A preliminary study of *Gonium viridistellatum* sp. nov. (Chlorophyta, Volvocaceae). Bull Jpn Soc Phycol. 1977;25 (supplement: A Memorial Issue Honoring the Late Professor Yukio Yamada): 379–384.

Table S2. Specific primers used for genomic PCR for strains of *Gonium pectorale* (Additional file 1: Table S1)

Gene (Accession No.)	Primer designation	Sequence (5'-3')
MID (LC062718)	GpMID-F1	CACGCCCATATCTTGGGGTCGGGGTCGG
	GpMID-R1 [a]	CCTTGCGATACGGCCAACGGGGAATGCCC
FUS1 (LC062719)	GpFUS1-F2	CGGCCTACGGCTACATGAATGGCATTCTCC
	GpFUS1-R2 [a]	GCCACAATTCCCCGCCACCTCCGTTTGCG
Actin (AB973307)	GpACT-F1	CACCTCTTACAATGGCCGAGGAGGGCGAGG
	GpACT-R1 [a]	CGGCATGCTGCGGCGACCAGACGCACC

[a] Reverse primer.

Table S3. Recovery results of vegetative cells of *Gonium pectorale* strain NIES-4502 after possible optimal cryogenic treatments (6% DMF; Table 1) in liquid nitrogen by using a simple cryopreservation module (Thermo Scientific™ Mr. Frosty™ Freezing Container [a], Thermo Fisher Scientific, Waltham, MA, USA) for two-step cooling in cryopreservation [b].

Conditions for cryopreservation	Total viability (range)[%]	MPN cell numbers in three tubes (/mL) (control) [c]
0.2 mL 6% DMF in 0.2 mL PCR tube	0 (0-0)	0, 0, 0 (180000)
1.0 mL 6% DMF in 2 mL cryotube	0.0013 (0-0.0040)	7.2, 0, 0 (180000)

[a] Designed to achieve a rate of cooling very close to -1°C/minute in a deep freezer.
[b] Protocols are the same as those described in Materials and Methods section in the main text except for using this simple module for the first step of two-step cooling in a deep freezer to reach approximately -80°C.
[c] Numbers corrected based on "0.05 mL of the sample plus 0.05 mL of VTAC medium" used for initial inoculation (see Methods).

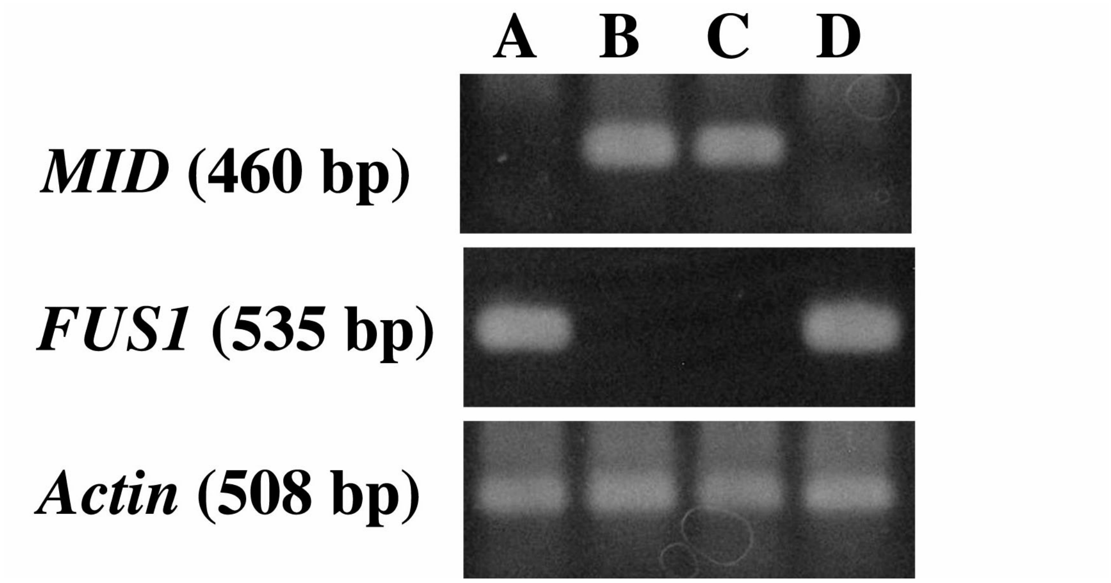

Figure S1. Mating type determination of four newly established strains of *Gonium pectorale* (NIES-4499−4502, Additional file 1: Table S1) by genomic PCR of mating type minus-specific minus dominance gene (*MID*) and mating type plus-specific gamete plasma membrane protein gene (*FUS1*). *Actin* is an autosomal gene (control). For primers used, see Additional file 1: Table S2. A. NIES-4499. B. NIES-4500. C. NIES-4501. D. NIES-4502. For full-length original gel images, see Additional file 1: Figure S2.

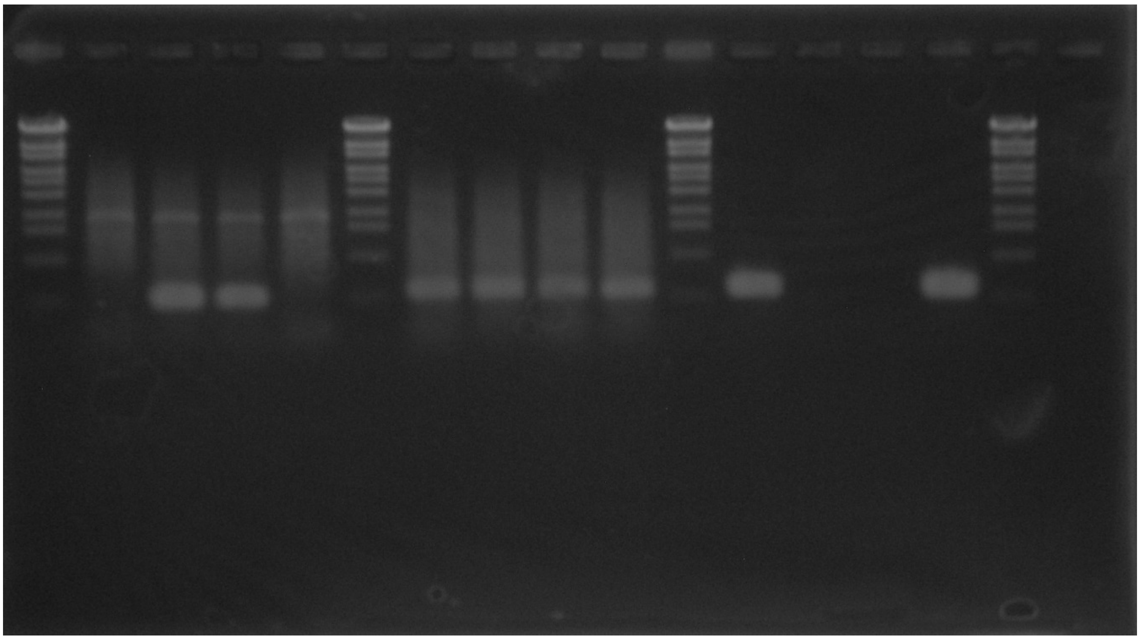

Figure S2. Full length, unprocessed gel images of the three genes shown in Additional file 1: Figure S1. Marker 6（λ/StyI digest）marker (NIPPON GENE, Tokyo, Japan) was used as a molecular size marker (1st, 6th, 11th and 17th lanes). 2nd, 3rd, 4th and 5th lanes: A, B, C and D, respectively, of *MID* (Additional file 1: Figure S1). 7th, 8th, 9th and 10th lanes: A, B, C and D, respectively, of *Actin* (Additional file 1: Figure S1). 12th, 13th, 14th and 15th lanes: A, B, C and D, respectively, of *FUS1* (Additional file 1: Figure S1).
